# Kisspeptin-10 Modulates the Proliferation and Differentiation of the Rhesus Monkey Derived Stem Cell Line: R366.4

**DOI:** 10.1155/2013/135470

**Published:** 2013-11-28

**Authors:** Tanzeel Huma, Zhengbo Wang, Joshua Rizak, Fiaz Ahmad, Muhammad Shahab, YuanYe Ma, Shangchuan Yang, Xintian Hu

**Affiliations:** ^1^State Key Laboratory of Brain and Cognitive Sciences, Kunming Institute of Zoology, Chinese Academy of Sciences, Kunming, Yunnan 650223, China; ^2^Reproductive Neuroendocrinology Laboratory, Department of Animal Sciences, Faculty of Biological Sciences, Quaid-i-Azam University, Islamabad 45320, Pakistan; ^3^State Key Laboratory of Crop Genetics and Germplasm Enhancement, Nanjing Agricultural University, Nanjing 210095, China

## Abstract

The rhesus monkey embryonic stem cell line R366.4 has been identified to differentiate into a number of cell types. However, it has not been well characterized for its response to drugs affecting reproductive endocrinology. Kisspeptins (KPs) are ligands for the GPR-54, which is known to modulate reproductive function. The current study was designed to determine the effect of the KP-10 peptide on R366.4 cells and to investigate the role of KP-GPR54 in the cell proliferation process. Four different doses (0.1, 1, 10, and 100 nM) of KP-10 and control were selected to evaluate cell growth parameters and cellular morphological changes over a 72 hr period. The cells were treated with kisspeptin-10 during the early rosette stage. Proliferation rates, analyzed by flow cytometry and cell count methods, were found to be decreased after treatment. Moreover, the number of rosettes was found to decrease following KP-10 treatments. Morphological changes consisting of neuronal projections were also witnessed. This suggested that KP-10 had an antiproliferative effect on R366.4 cells leading to a differentiation state and morphological changes consistent with neuronal stem cell development. The R366.4 stem cell line differentiates based on kisspeptin signaling and may be used to investigate reproductive cell endocrinology *in vitro*.

## 1. Introduction

Embryonic stem cells (ESC) are capable of self-renewal and differentiation into cells of the three germ cell layers [1–4]. This includes the differentiation into various cell types, such as hematopoietic, epithelial, neuronal, vascular, cardiac muscles, smooth muscles, chondrogenic, and adiponergic lineages [5]. ESCs have been derived/isolated from mouse embryos [1, 2], nonhuman primates [6], and humans [7]. As such, ESCs have become a valuable molecular biology research tool as they allow for a number of studies into embryonic development toxicology and pharmacology [8, 9].

At present, 26 ESC lines from the rhesus macaque, an animal often used as a model to study human disease, have been produced from *in vivo* flushed blastocysts [7, 9]. Among these are eight R series cell lines (4 males and 4 females) made from blastocysts produced* in vivo* by Dr. James Thomson at the Wisconsin National Primate Research Center [6]. This work identified one particular cell line, the R366.4 cell line, which has the differentiation potential to form teratomas, embryoid bodies, neuronal progenitor cells, and cells with glial and neuronal phenotypes [10]. However, the R366.4 cell line has not been well characterized for physiological changes in response to drug treatments.

Kisspeptins (KP) are peptides that have the potential for therapeutic use [11] which are expressed by the tumor melanoma cell metastasis suppressor gene KiSS1 [12] and have a major role in reproduction and metabolic regulation [13–15]. Kisspeptin peptides are endogenous ligands for the G-protein coupled receptor GPR54, also known in the literature as hOT7T175 or AXOR12 [16–18]. The C-terminal end of the kisspeptin peptides binds and activates GPR54 signaling [17, 19], which has a number of downstream effects. Of note, kisspeptin/GPR54 signaling appears to be involved in cell growth and differentiation. For example, kisspeptin treatments have been reported to play a role in GnRH neutrite growth *in vitro* [20]. However, the activation of GPR54 by KP has been shown to inhibit cell motility, proliferation, invasion, chemotaxis, and metastasis [16, 17]. This complicates the role of kisspeptin signaling in stem cell growth and differentiation. As such, the effect of kisspeptin on the monkey R366.4 stem cell is unknown.

To evaluate R366.4 stem cell differentiation in response to KP drug treatments, normal pluripotent R366.4 cells were treated with kisspeptin-10 to measure the proliferation, differentiation, and morphological changes to the cells.

## 2. Materials and Methods

### 2.1. Rhesus Monkey Embryonic Stem Cells

R366.4 rESCs were kindly provided by Dr. James A. Thomson at The Wisconsin Regional Primate Research Center, University of Wisconsin, USA. The cells were cultured on a feeder layer of irradiated monkey ear skin fibroblasts (MESFs) from a 1-week-old rhesus monkey in ESC culture medium [6]. The embryonic stem cell culture media contained 85% DMEM (Gibco), 15% Fetal Bovine Serum (FBS) (Invitrogen China Limited, Beijing, China), 2 mM glutamine (Sigma-Aldrich China Inc., Shanghai, China), 0.1 mM nonessential amino acids (Sigma-Aldrich China Inc., Shanghai, China), 50 *µ*g/mL_Penicillin-Streptomycin-Glutamine (PSG) (Sigma-Aldrich China Inc., Shanghai, China) and 0.1 M *β*-Mercaptoethanol (*β*-ME).

### 2.2. Formation of Embryoid Bodies (EBs)

**Figure 1:**
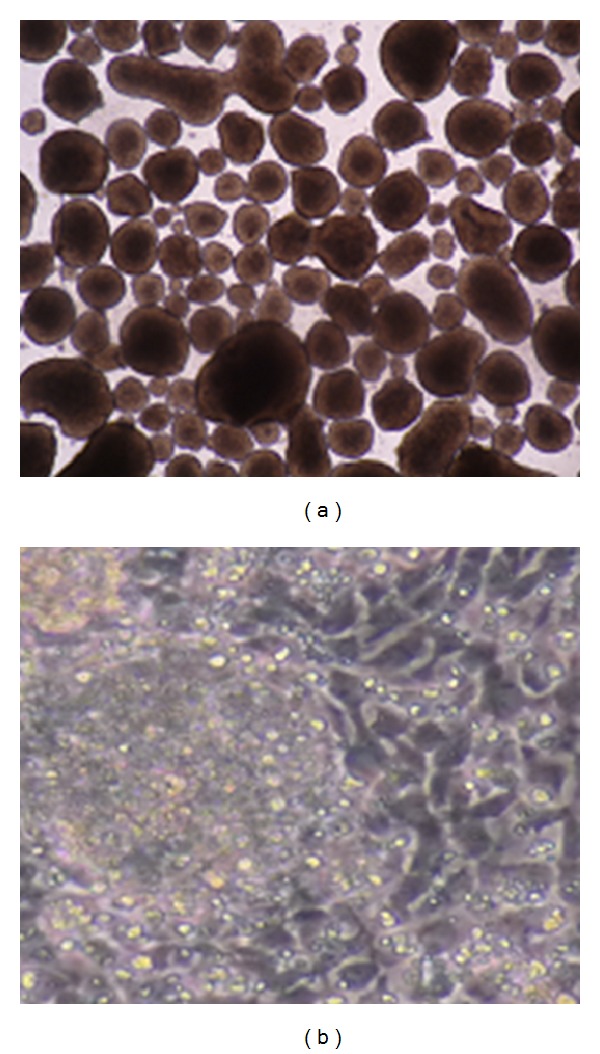
(a) Embryoid bodies (EBs). (b) Early rosette stage. The drug, kisspeptin-10, was added to this stage.

ESC colonies were digested with dispase (Invitrogen China Limited, Beijing, China; 1 mg/mL) at 37°C for 5–8 minutes, washed with PBS (Invitrogen China Limited, Beijing, China) to remove dispase, suspended in the N/M medium (1 : 1 DMEM/F12 (Gibco, Invitrogen China Limited, Beijing, China) : Neural Basal Medium (Gibco, Invitrogen China Limited, Beijing, China) containing 1xN2 supplement, 1xB27 (Gibco, Invitrogen China Limited, Beijing, China), and 2 mM glutamine (Sigma-Aldrich China Inc., Shanghai, China). Cells were plated on 15 × 30 mm wells coated with agar (A1296-500G, Sigma-Aldrich China Inc., Shanghai, China) and allowed to aggregate for 4 days to form EBs (Figure 1(a)).

### 2.3. Neural Progenitor Cell Induction

After aggregation, EBs were selected and cultured in Neuronal Progenitor Media (1 : 1 DMEM/F12: containing 2 *μ*g/mL heparin and 2 mM glutamine) in 4 well plates coated with Extracellular Matrix Media (ECM, Sigma-Aldrich China Inc., Shanghai, China) for 3 to 4 days till rosettes appeared (Figure 1(b)). Rosette-Neuronal Stem Cells (R-NSC) represent the first characterized neuronal stem cell stage capable of responding to patterning cues that direct differentiation to different specific regions [21].

### 2.4. Kisspeptin-10 Treatment

On day 12 (early rosettes stage—Figure 1(b)), the cells were treated with vehicle (ddH_2_O), 0.1 nM, 1 nM, 10 nM, and 100 nM kisspeptin-10 (Phoenix Biotech Co., Ltd., Beijing, PR China). The dose was decided based on previous studies [20]. The media were changed every day and fresh drug was added immediately after changing media. All experiments were repeated in triplicate.

### 2.5. Flow Cytometry

The cells treated with the four different doses of kisspeptin-10 mentioned above were then harvested after 72 hrs, washed with phosphate-buffered saline (PBS) three times, and fixed with 700 mL/L ethanol at 4°C overnight. Fixed cells were washed three times with PBS and stained with 800 mL propidium iodide (Sigma-Aldrich China Inc., Shanghai, China) and 200 mL deoxyribonuclease-free ribonuclease A (Sigma-Aldrich China Inc., Shanghai, China) in PBS. The fluorescence intensity of propidium iodide-stained nuclei was detected with a flow cytometer (EPICS-XL, Coulter, USA).

### 2.6. Cell and Rosette Count

Rosettes were counted manually every 24 hrs up to 72 hrs after kisspeptin-10 treatments under the light microscope (CKX41; Olympus, Tokyo, Japan) at 10x magnification. Cells were counted at the same time points using the haemocytometric method at 20x magnification under the light microscope (CKX41; Olympus, Tokyo, Japan).

### 2.7. Tunnel Assay

Cells were fixed in 24 well plates with 4% paraformaldehyde and apoptosis was measured with the In Situ Cell Death Detection Kit POD (supplemented with 0.1% sodium citrate), according to the manufacturer's instructions (Roche, NJ, USA). Cells were exposed to DNase I 10 min prior to the assay (BioLabs Inc. NE) to provide a positive control. Terminal transferase was omitted to provide a negative control. Tunnel positive and the total number of cells were counted at four randomly selected locations in each plate well. The percentage of tunnel positive to total cell count was calculated.

### 2.8. Morphological Changes

The cells were critically observed on a daily basis for axonal growth, general shape, and the development of colonies under the light microscope (CKX41) at 10x and 20x magnifications. Morphological changes were recorded on images taken with a Canon PowerShot ELPH 330 HS camera (Tokyo, Japan).

### 2.9. Statistics

Repeated measured one way ANOVAs followed by Tukey's posttest were performed for data analysis using the GraphPad 5 software (GraphPad Software, Inc., La Jolla, USA). Significance was set at *P* < 0.05.

## 3. Results

### 3.1. R366.4 Cell Growth and Development

The R366.4 cell line was found to grow normally on irradiated MESFs in ESC culture medium. Embryoid bodies began to form after 3 to 4 days, which were then transferred to ECM media until rosettes appeared. At which time, KP-10 treatments were initiated.

### 3.2. Effect of KP-10 on R366.4 Cell Proliferation

**Figure 2:**
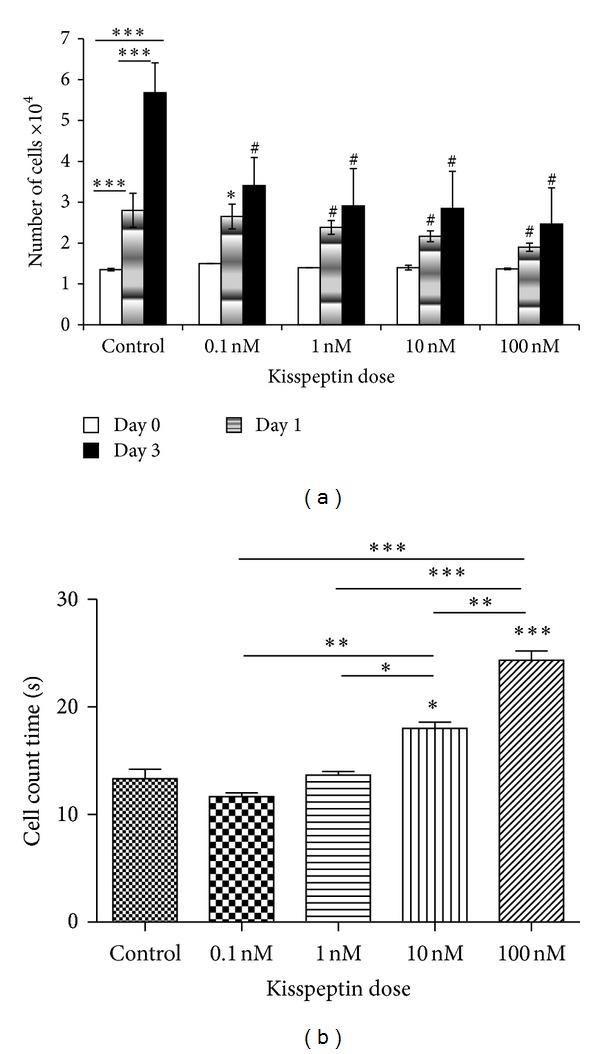
(a) The effect of kisspeptin dose and time on the proliferation rate of R366.4 cells. Bars indicate the mean number of cells ± SEM. ∗∗∗ represents comparison to day 0 cell count, and ∗ and # represent same day comparisons to control counts. (b) Flow cytometry of R366.4 cells following 72 hrs of KP-10 treatment results. The *y*-axis represents the time it takes to count 10,000 cells. Significance was observed at *P* < 0.0001 in a repeated measure ANOVA followed by Tukey's posttest. ∗∗∗, # represents the *P* < 0.0001; ∗∗ represents *P* < 0.001; ∗ represents *P* < 0.01.

Different doses of KP-10 were used to treat R366.4 cells. A significant decrease (*P* < 0.0001) in the proliferation of the cells was observed. Significant decreases were seen after 3 days of KP-10 treatment (*P* < 0.0001) in comparison to control after 3 days (Figure 2(a)). The numeric values are given in Table 1. Flow cytometry was performed after the 3-day treatment of different doses of KP-10. The proliferation rate was found to be decreased and a highly significant decrease in proliferation was observed at 100 nM treatment (*P* < 0.0001) in comparison to lower doses and control (Figure 2(b)).

### 3.3. Effect of KP-10 on R366.4 Rosette Formation

**Figure 3:**
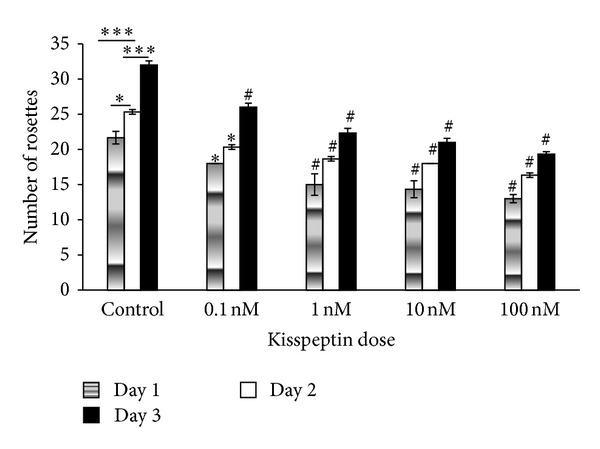
The effect of KP-10 on R366.4 rosette formation. A repeated measure ANOVA followed by Tukey's posttest. KP-10 dose showed a significant decrease in the number of rosettes (*P* < 0.0001) compared to controls. ∗∗∗ represents control group comparisons(*P* < 0.0001), ∗ indicates *P* < 0.01, ^#^ *P* < 0.0001, respectively.

During neuronal differentiation, embryonic stem cells undergo morphological changes characterized by the formation of radially organized columnar epithelial cells called neural rosettes [22, 23]. Cells have a wide differentiation potential solely during the early rosette stage which is lost upon further proliferation *in vitro* [24]. Different doses of kisspeptin-10 were found to decrease the number of rosettes of R366.4 cells. At higher doses (100 nM) the total number of rosettes significantly decreased (*P* < 0.0001) compared to controls. Increasing the treatment time was found to significantly reduce the number of rosettes (*P* < 0.001; Figure 3, Table 2).

### 3.4. Effect of KP-10 on R366.4 Cell Apoptosis

KP-10 treatment was not found to significantly affect R366.4 cell apoptosis. There was nonsignificant evidence that KP-10 might reduce apoptotic indicators in the cell line (data not shown); however, this observation did not reflect a consistent trend in respect to the drug treatment.

### 3.5. Effect of KP-10 on R366.4 Cells Morphology

**Figure 4:**
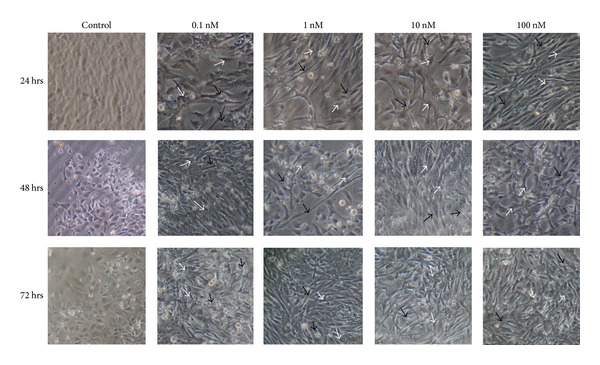
R366.4 ESC morphology following KP-10 treatments. R366.4 cell bodies change shape and size (white arrow) leading to dendritic extensions and cell-to-cell interactions (black arrows) with increasing KP-10 treatments.

Morphological changes were observed daily to assess the Effect of KP-10 treatment on cell structure. All four KP-10 doses were observed to affect the morphology of the growing neuronal stem cells in comparison to controls (Figure 4). Cell body shape changed to an elongated form like a pendulum. The dendritic extensions or/and stress fibers were longer and larger and had an increased number of cell-to-cell interactions after KP-10 treatments (Figure 4). These changes were more pronounced with higher KP-10 treatment doses. This indicated that KP-10 may lead to the differentiation of neuronal stem cells.

## 4. Discussion

Rhesus monkey ESCs (rESCs) have become an important tool used in molecular biology research. They can be used to explore complicated disease mechanisms *in vitro* [25, 26] and can also be used in transplantation studies to model diseases *in situ* [3]. One of the great advantages of rhesus monkey ESCs is that they share many characteristics with human ESCs, including cellular morphology, surface marker expression, and developmental potential [10], as well as greater than 90% DNA homology [27–30], which allows for better mimicry of the human system. However, the full potential of rESCs use has not been met, as many cell lines, like the R366.4 cell line, have not been fully characterized with respect to proliferation, differentiation, and response to specific drug treatments.

In this study, the proliferation, differentiation, and morphology of r336.4 cells were evaluated after kisspeptin-10 treatments. KP-10 is a small peptide that has been identified as a potential therapeutic in the treatment of reproductive dysfunction. A better understanding of KP-10 signaling on r336.4 cell growth will provide new avenues into drug research, reproductive dysfunction pathology, and stem cell transplantation functionality.

KP-10 was found to decrease R366.4 cell number (proliferation) at different doses of KP-10 (0.1, 1.0, 10.0, and 100 nM). This suggested that kisspeptin might play an antiproliferative role in growing R366.4 embryonic stem cells. KP-10 treatments also reduced the formation of rosettes in a dose dependent manner, which suggested kisspeptin may induce differentiation of R366.4 cells into specific cell types. Although the specific type of cell being form was beyond the scope of this study, the cells were found to undergo a morphological change following KP-10 treatments. At the highest dose of KP-10 (100 nM), the cell bodies were larger in comparison to cells treated with other doses and neuronal fibers and/or extensions (Figure 4; black arrows) formed in response to KP-10, leading to cell-to-cell interactions. Unfortunately the specific type of these fibers and/or extensions is not clear and requires further study.

As R366.4 cells are pluripotent cells lines [6] that are responsive to KP-10, this suggests the cells may have endogenous expression of GPR54 (the kisspeptin receptor). Kisspeptin has been found to have inhibited cell proliferation in a previous study. However, this inhibition was only witnessed in cells transfected with a GPR54 expressing plasmid (leading to higher GPR54 expression) [31]. Although the cells used in the Ziegler et al. study endogenously expressed GPR54, kisspeptin treatment did not have an antiproliferative effect without the GPR54 plasmid, suggesting that KP-10 antiproliferation is dependent on high levels of GPR54 expression. It is possible that GPR54 receptor expression was increased under the effect of the KP-10 signaling, as kisspeptin has been shown to increase GPR54 expression [32]. Although it is not known whether the R366.4 cell lines express GPR54, it is likely that the R366.4 neuronal stem cells endogenously express GPR54 receptors, despite needing further study to confirm.

Interestingly, apoptotic markers in the R366.4 cells were not found after KP-10 treatment. Kisspeptin/GPR54 signaling had been reported not only to be proapoptotic in most studies, as it has been found to suppress the Akt pathway [33], activate the RhoA-Rock/RhoA pathway, and cause apoptosis in a breast cancer the HEK-236 cell line [34], but also to be anti apoptotic in others, as it has been found to inhibit TNF alpha induced Rho signaling, thereby inhibiting apoptosis [35]. This may suggest that the role of KP-10 in apoptosis may be stage specific. It is not known if kisspeptin inhibits TNF alpha induced Rho signaling in the R366.4 cells lines to inhibit apoptosis, but it is possible that kisspeptin/GPR54 acts stage specifically to modulate cell growth and differentiation.

Wide cellular differentiation potentials are proxy to the early rosettes stage, and differentiation is lost or decreased if the rate of proliferation increases [24]. At higher doses of KP-10, a lower rate of cell proliferation was observed as well as marked decrease number of rosette formation. This suggests that the cells had begun to differentiate in response to kisspeptin signals. It is not known whether kisspeptin modulates the Notch pathway and Sonic Hedgehog (SHH) signaling, which are both necessary to maintain the R-NSC stage. It has been found that in the absence of these pathways, NSC decrease rosette numbers and form more specific neuronal cell types [36]. These findings are consistent with the observations presented here and the morphological changes witnessed in the R366.4 cell line, which suggest that kisspeptin may modulate the Notch Pathway and/or a similar signaling cascade during development.

The findings in this study, that R366.4 cell growth, differentiation, and neuronal fiber formation are modulated by KP-10 treatments, suggest that this stem cell line may be used further to investigate reproductive cell endocrinology *in vitro*, as well as having further implications in future stem cell therapies that can be targeted directly to reproductive development. At this time, however, the specific differentiation patterns observed following KP-10 treatments require further study and a much more detailed investigation to identify the molecular details behind the R366.4 cell morphological changes. Nonetheless, the work performed in this study provides a primer into the further development and use of the R366.4 cell line in biomedical research.

## 5. Conclusion

The growth and differentiation of the R366.4 stem cell line is modulated by reproductive endocrine kisspeptin. Kisspeptin-10 inhibits the proliferation of R366.4 stem cells without causing apoptosis. This suggests that the R366.4.4 cell line may endogenously express GPR54 (the kisspeptin receptor). Decreases in the number of neuronal rosettes, which are the hallmark of neuronal differentiation, indicated that most of the cells had differentiated to specific cell types. Furthermore, the shape and size of R366.4 cell bodies changed after KP-10 treatment.

## Figures and Tables

**Table 1:** Presenting the mean value of number of cells ×10^4^ ± SEM.

Treatment	Day 0	Day 1	Day 3
Control	1.35 ± 0.05	2.8 ± 0.41	5.68 ± 0.7
0.1 nM	1.5 ± 0	2.65 ± 0.30	3.408 ± 0.6
1 nM	1.4 ± 0	2.38 ± 0.16	2.90 ± 0.9
10 nM	1.4 ± 0.051	2.16 ± 0.13	2.85 ± 0.9
100 nM	1.37 ± 0.01	1.9 ± 0.1	2.46 ± 0.8

**Table 2:** Presenting the mean value of number of rosettes ± SEM.

Treatment	Day 1	Day 2	Day 3
Control	21.6 ± 0.8	25.3 ± 0.30	32 ± 0.5
0.1 nM	18 ± 0 0	21.3 ± 0.30	26 ± 0.5
1 nM	15 ± 1.5	18.61 ± 0.30	22.3 ± 0.6
10 nM	14 ± 1.2	18 ± 0	21 ± 0.5
100 nM	13 ± 0.51	16.3 ± 0.30	19.3 ± 0.3
